# Flowering and Growth Responses of Cultivated Lentil and Wild *Lens* Germplasm toward the Differences in Red to Far-Red Ratio and Photosynthetically Active Radiation

**DOI:** 10.3389/fpls.2017.00386

**Published:** 2017-03-21

**Authors:** Hai Y. Yuan, Shyamali Saha, Albert Vandenberg, Kirstin E. Bett

**Affiliations:** Department of Plant Sciences, University of Saskatchewan, SaskatoonSK, Canada

**Keywords:** lentil, red/far-red ratio, photosynthetically active radiation, photomorphogenesis, photosynthesis

## Abstract

Understanding environmental responses of pulse crop species and their wild relatives will play an important role in developing genetic strategies for crop improvement in response to changes in climate. This study examined how cultivated lentil and wild *Lens* germplasm responded to different light environments, specifically differences in red/far-red ratio (R/FR) and photosynthetically active radiation (PAR). Three genotypes of each the seven *Lens* species were grown in environmentally controlled growth chambers equipped to provide light treatments consisting of different R/FR ratios and PAR values. Our results showed that overall, days to flower of *Lens* genotypes were mainly influenced by the R/FR induced light quality change but not by the PAR related light intensity change. The cultivated lentil (*L. culinaris*) showed consistent, accelerated flowering in response to the low R/FR light environment together with three wild lentil genotypes (*L. orientalis* IG 72611, *L. tomentosus* IG 72830, and *L. ervoides* IG 72815) while most wild lentil genotypes had reduced responses and flowering time was not significantly affected. The longest shoot length, longest internode length, and largest leaflet area were observed under the low R/FR low PAR environment for both cultivated and wild lentils. The distinctly different responses between flowering time and elongation under low R/FR conditions among wild *Lens* genotypes suggests discrete pathways controlling flowering and elongation, which are both components of shade avoidance responses. The yield and above-ground biomass of *Lens* genotypes were the highest under high R/FR high PAR conditions, intermediate under low R/FR low PAR conditions, and lowest under high R/FR low PAR light conditions. Three *L. lamottei* genotypes (IG 110809, IG 110810, and IG 110813) and one *L. ervoides* genotype (IG 72646) were less sensitive in their time to flower responses while maintaining similar yield, biomass, and harvest index across all three light environments; these are indications of better adaptability toward changes in light environment.

## Introduction

Light is essential for plant growth and development. It is a source of energy for photosynthesis and provides information for regulation of growth and development (Smith, 1982). Light is necessary for the adaptation of plants to specific environments. Information based on light quality induces a collective photomorphogenetic response (Quail, 2002), which is controlled by several photoreceptors including blue-light absorbing phototropin and cryptochrome and the red and far-red light absorbing phytochrome family (Rockwell et al., 2006; Möglich et al., 2010). Among these photoreceptors, phytochromes play a key role in photomorphogenesis and control about 10% of the plant transcriptome (Quail, 2002). Phytochromes exists in two forms, the red-light absorbing Pr, which absorbs maximally at 660 nm and is generally considered to be biologically inactive, while the far-red light absorbing Pfr, which absorbs maximally at 730 nm and is biologically active (Franklin and Whitelam, 2005). Absorption of light by either Pr or Pfr results in phototransformation between these two forms, which drives the on/off switching of the successive signaling pathway (Han et al., 2007).

Green plants selectively absorb blue and red wavelengths through chlorophyll and carotenoid photosynthetic pigments. The radiation reflected by green leaves is relatively enriched in the far-red spectrum (Smith and Whitelam, 1997). A resulting decrease in red/far-red (R/FR) ratio in the surrounding environment is sensed by the phytochromes, thereby signaling the presence of neighboring plants as potential competition. This leads to enhanced stem elongation and accelerated transition to flowering, which are features of the shade avoidance response (SAR) (Whitelam and Devlin, 1997). A reduction in light intensity usually triggers a similar SAR (Ballare et al., 1991). Under natural conditions, the SAR allows plants to compete with neighboring vegetation for limited resources (Schmitt et al., 2003). For crop species, however, SAR could lead to decreased yield if plants spend resources on vegetative growth at the expense of reproductive development (Board, 2000; Kebrom and Brutnell, 2007; Casal, 2013). Stem elongation often leads to lodging, as observed by Gong et al. (2015) in soybean (*Glycine max*), and genotypes displaying reduced SAR are suggested to have improved performance at higher plant densities (Kebrom and Brutnell, 2007; Gong et al., 2015).

A close relationship between light intensity and plant growth and yield has been described for many plant species, including legumes (Bethlenfalvay and Phillips, 1977; Baligar et al., 2008; Mielke and Schaffer, 2010). The literature contains a few reports on flowering and growth of legume species in response to light quality changes (Board, 2000; Heraut-Bron et al., 2000; Gong et al., 2015), but little published information exists on the interactive effects of light intensity and light quality.

Lentil (*Lens culinaris* Medik.) is a quantitative long-day species (Summerfield et al., 1985). Lentil plays a significant role in supporting environmentally sustainable agriculture through its nitrogen fixation ability, and is internationally recognized as part of the solution to global food and nutritional insecurity. The genus *Lens* has seven species that originated around the Mediterranean and into the Middle East and the domesticated lentil is *L. culinaris* (Cubero et al., 2009). The other six wild species within the genus *Lens* are *L. culinaris, L. orientalis, L. tomentosus, L. odemensis, L. lamottei, L. ervoides*, and *L. nigricans* (Wong et al., 2015). Resistance to biotic or abiotic stresses that are lacking in cultivated lentil have been identified in wild relatives (Tullu et al., 2006, 2010; Fiala et al., 2009; Podder et al., 2012; Vail et al., 2012) and this genetic resource is considered to have great value for future genetic improvement.

Growth and development of cultivated lentil was significantly affected when standard lighting (fluorescent and incandescent bulbs) was replaced with high efficiency and high output fluorescent bulbs in our controlled environment growth chambers. Flowering of cultivated lentil was delayed by up to 30 days (Mobini et al., 2012, 2016; Yuan et al., 2015) but some wild germplasm seemed unaffected. The new light system affected both light quality (increased R/FR) and light intensity [increased photosynthetically active radiation (PAR)]. As a first step to gaining an understanding of the genetic basis of light responses in genus *Lens*, we used a collection of wild and cultivated *Lens* germplasm to characterize the flowering and growth responses to changes in R/FR and PAR. The hypothesis was that flowering and growth development in *Lens* germplasm could be different due to the differences in R/FR and PAR and if so, these genotypes would represent key genetic resources for developing lentil cultivars with better adaptation to variable light environments.

## Materials and Methods

### Plant Materials

Three genotypes of each of the seven *Lens* species were selected from germplasm currently in use in the breeding program at the Crop Development Centre (CDC), University of Saskatchewan (USASK) (**Table 1**). Prior to planting, seeds of all genotypes were stored at -20°C for 1 week and then scarified to improve imbibition and germination. Square 10-cm pots were filled with growth medium consisting of 50% Sunshine^®^ Mix #3 and 50% Sunshine^®^ Mix #4 (Sun Gro Horticulture Canada, Ltd, Seba Beach, AB, Canada^1^). Two seeds were sown in each pot and thinned to a single plant after emergence. Each repeat had four pots (replicates) per genotype and the experiment was repeated once. All plants were grown and maintained in controlled growth cabinets set at 22°C/16 h day and 16°C/8 h night for both repeats.

**Table 1:** Lentil species and genotypes used in light experiments.^1^

Species	Genotypes	Country of origin	Geolocation (lat, long)	Elevation (m)	Seed source
*L. culinaris*	Eston	Canada	52.8, -106.4	481	CDC, USASK
	CDC Greenstar	Canada	52.8, -106.4	481	
	CDC QG-2	Canada	52.8, -106.4	481	
*L. orientalis*	IG 72529	Turkey	38.7, 39.2	1132	ICARDA
	IG 72611	Turkey	38.7, 42.2	2117	ICARDA
	BGE 016880	Israel	N/A	N/A	Universidad de León^2^
*L. tomentosus*	IG 72614	Turkey	37.9, 40.3	662	ICARDA
	IG 72805	Turkey	37.8, 39.8	1150	ICARDA
	IG 72830	Turkey	37.7, 39.2	1250	ICARDA
*L. odemensis*	IG 72623	Turkey	37.4, 41.0	1001	ICARDA
	IG 72639	Syria	34.8, 36.4	950	ICARDA
	IG 72760	Syria	32.7, 36.6	1100	ICARDA
*L. lamottei*	IG 110809	Spain	36.8, -5.7	150	ICARDA
	IG 110810	Spain	36.8, -5.4	800	ICARDA
	IG 110813	Spain	37.4, -4.3	660	ICARDA
*L. ervoides*	IG 72646	Syria	35.8, 36.0	570	ICARDA
	IG 72815	Turkey	37.6, 36.5	860	ICARDA
	L01-827A	Jordan^3^	30.8, 35.6^3^	1250^3^	CDC, USASK^4^
*L. nigricans*	IG 116024	Turkey	37.8, 29.1	560	ICARDA
	IG 136640	Italy	45.1, 7.1	1464	ICARDA
	IG 72555	Spain	37.3, -2.5	1314	ICARDA


*^1^Information in this table was extracted from Genesys (https://www.genesys-pgr.org/welcome). ^2^Fratini et al. (2007). ^3^Geolocation and elevation are from *L. orientalis* IG 72847. ^4^Fiala et al. (2009); L01-827A is a single plant selection from a packet of *L. orientalis* IG 72847 from ICARDA.*

### Light Environment and Growing Conditions

**FIGURE 1:**
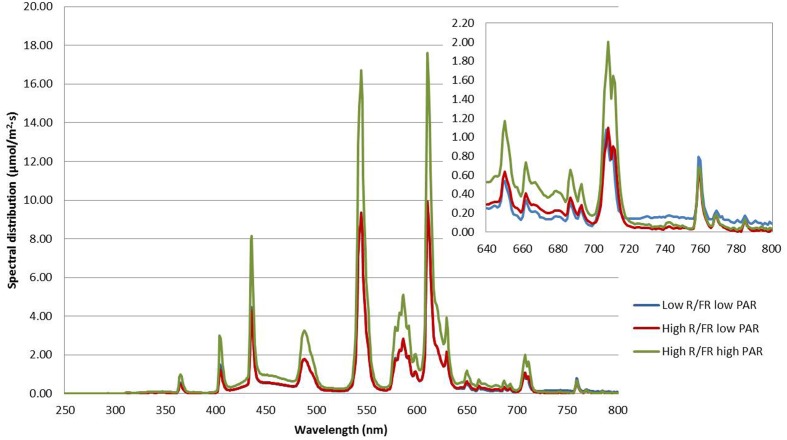
**Spectral distribution and characteristics of the light treatments used to compare the effect of light quality and quantity on lentil growth and development.** The upper inset shows an enlarged version of the 640–800 nm region.

Two Conviron GR48 walk-in plant growth chambers and one Conviron PGV36 walk-in plant growth chamber were used in the experiment. The light properties of the three growing environments are described in **Table 2**. Spectral distribution and characteristics of the light treatments used in the experiment are shown in **Figure 1**. The high R/FR was the natural ratio of the renovated light system in the growth chambers fitted with T5 841 High Output Fluorescence bulbs (Philips, Andover, MA, USA). The low R/FR environment was fitted with evenly spaced infrared bulbs (Model FLR48T5NIR-HO, DN Lighting, Co. Ltd, Hiratsuka City, Japan) to replace the T5 841 fluorescent bulbs in the light bank. The high PAR condition was the natural light intensity of the renovated light system while the low PAR condition was reached by adjusting the height of the light bank. The spectral photon flux and PAR of each light treatment was measured using a spectroradiometer (Apogee Instruments, Model PS-300, Logan, UT, USA). The R and FR values were calculated using the spectral photon flux at 650–670 and 720–740 nm, respectively (Smith, 1982).

**Table 2:** Light properties of the three growing environments used in the current experiment.

Treatments	Low R:FR Low PAR	High R:FR Low PAR	High R:FR High PAR
Red_650-670_/Far-red_720-740_ ratio	1.6	8.1	8.1
Photosynthetically active radiation (PAR, μmole/m^2^⋅s)	420	420	680


### Data Collection and Analysis

Lentil development stages were based on Erskine et al. (1990) with minor modifications to suit the wild lentil species. Days to flower were calculated based on days from emergence to R1 (one open flower at any node), and the node number of the first open flower was recorded. Leaflet area was measured using the leaflets produced at the R1 flowering node using a flatbed scanner (Epson Perfection V700 Photo Scanner, Epson Canada, Markham, ON, Canada) and WinFOLIA^TM^ software (Regent Instruments, Inc., Québec City, QC, Canada). Lentil plants produce one leaf per node, but have 5–15 leaflets per leaf depending on species and genotype. Five fully expanded leaflets beginning from the base of the leaf at the R1 flowering node from four plants (replicates) were removed from the plants and kept in Petri-dishes on top of ice and leaflet area was immediately measured. Shoot length and node number were recorded when the plants reached R5 (one mature pod at any node), at which point mesh bags were placed around the plants to collect seeds at maturity. Average internode length was calculated using node number at R5 divided by shoot length. The time from sowing to harvest was kept at 110 days, which corresponds to the regular growing period for field grown lentil in Western Canada. Above-ground plant materials were harvested separately for each plant and dried in the dryer. Above-ground biomass was recorded when plant materials were totally dried. Yield (seed production per plant) was recorded after threshing. Harvest index was calculated using the yield divided by the above-ground biomass. Data were collected from two repeats (four reps each), except for leaflet analysis, above-ground biomass, and harvest index which were recorded for the second repeat only. The General Mixed Model Procedure (PROC MIXED) in SAS (SAS Institute, Inc., Cary, NC, USA) was used to determine the effects of genotype, light treatment, and their interaction on observed traits. Paired comparisons were performed using Tukey’s honestly significant test, with *p*-values ≤ 0.05 considered significant.

## Results

### Overall Growth and Development in *Lens* Genotypes Subjected to Different Light Environments

Growth and development of each genotype varied among light environments. The light environment did not alter the time to emergence but affected the development time from VE (emergence) to R1 and from R1 to R5 in some genotypes (figure shown in Supplementary Materials). Typically, high R/FR delayed the development from VE to R1, while low PAR delayed the development from R1 to R5, if there was an effect. One extreme case was *L. ervoides* IG 72815, for which a mean 35-day difference from the VE to R5 stage was found between the low R/FR low PAR environment and the high R/FR low PAR environment. Of the three *L. nigricans* genotypes, two did not reach R1 under any light environment, while *L. nigricans* IG 72555 flowered after 94 days in the low R/FR low PAR environment. Therefore, these three genotypes were not included in further data analyses.

### Flowering in *Lens* Genotypes to Changes in Light Environment

**FIGURE 2:**
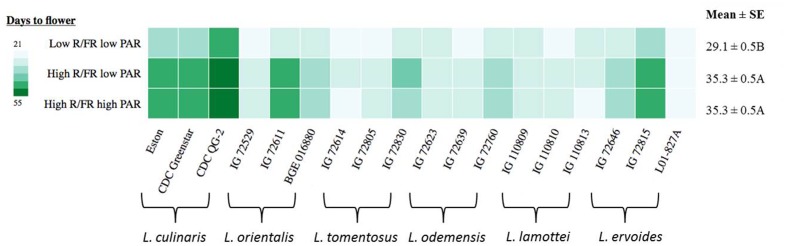
**Days to flower for three genotypes of each of six *Lens* species grown in light environments differing in red/far-red ratio (R/FR) and PAR.** Mean ± SE is from two repeats (eight replicates) and represents all *Lens* genotypes grown in the specific light environment. Different letters represent significant differences among treatments (Tukey’s test at *p* ≤ 0.05). Heat map is generated using the in-house developed computer program and the monochrome color coding system was used with the color from lighter to darker represented the values from lower to higher.

Days to flower (DTF) were significantly influenced by the genotype, the light environment, and the interaction between them (**Table 3**). Overall, significant differences were noted between low R/FR and high R/FR environments, while no difference in DTF was detected between low PAR and high PAR when grown under high R/FR conditions (**Figure 2**). Days to flower in cultivated lentil genotypes (*L. culinaris*: Eston, CDC Greenstar, and CDC QG-2) were all significantly (*p* ≤ 0.05) affected by the R/FR induced light quality change but not the PAR related light intensity change. Days to flower in wild lentil species was affected less by changes in either light quality or light intensity; the exceptions were *L. orientalis* IG 72611, *L. tomentosus* IG 72830, and *L. ervoides* IG 72815 for which flowering was significantly (*p* ≤ 0.05) delayed under the high R/FR environment like the cultivated lentil.

**Table 3:** Results of the two-way ANOVA for growth characteristics of *Lens* genotypes grown under three different light regimes (treatment).

Growth characteristic	Genotype		Treatment		Genotype × Treatment	
			
	F	Pr > F	F	Pr > F	F	Pr > F
Days to flower	65.32	<0.0001	81.11	<0.0001	2.53	<0.0001
Shoot length	19.53	<0.0001	291.60	<0.0001	2.07	0.0006
Internode length	12.29	<0.0001	271.95	<0.0001	1.21	**0.2033**
Leaflet area	31.47	<0.0001	73.39	<0.0001	5.97	<0.0001
Yield	7.30	<0.0001	73.72	<0.0001	1.96	0.0021
Biomass	7.64	<0.0001	85.46	<0.0001	1.60	0.0297
Harvest index	22.75	<0.0001	74.47	<0.0001	3.07	<0.0001


*Non-significant factors are bolded.*

### Shoot Length, Internode Length, and Leaflet Area of *Lens* Genotypes to Changes in Light Environment

**FIGURE 3:**
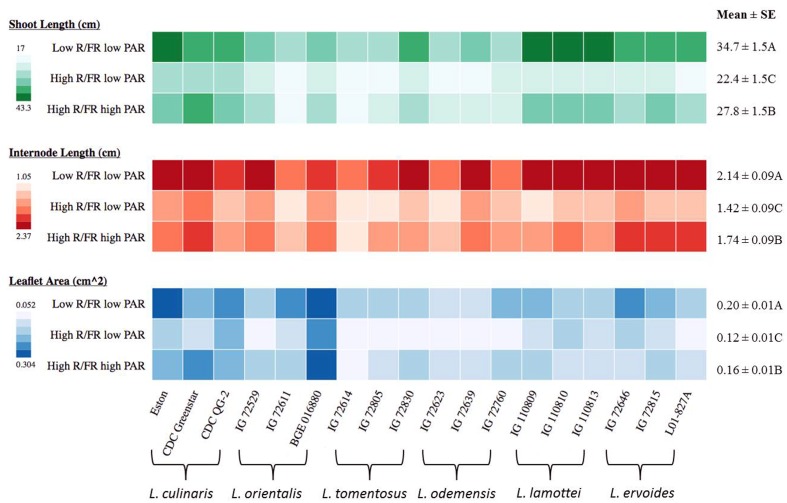
**Shoot length, internode length, and leaflet area for three genotypes of each of six *Lens* species grown in light environments differing in red/far-red ratio (R/FR) and photosynthetically active radiation (PAR).** Mean ± SE is from two repeats (eight replicates) for shoot length and internode length while one repeat (five replicates) for leaflet area and represents all *Lens* genotypes grown in the specific light environment. Different letters represent significant differences among treatments (Tukey’s test at *p* ≤ 0.05). Heat map is generated using the in-house developed computer program and the monochrome color coding system was used for each trait with the color from lighter to darker represented the values from lower to higher.

Both shoot length and internode length of *Lens* genotypes were significantly influenced by genotype, the light environment, and the interaction between them, except for the genotype × treatment interaction on internode length (**Table 3**). Significant differences were noted among species for shoot length and internode length and these differences were more distinct under low R/FR conditions (**Figure 3**). Low R/FR light resulted in significantly longer shoot and internode length compared to the high R/FR environment (*p* ≤ 0.05, **Figure 3**). Under high R/FR condition, high PAR resulted in significant longer shoots and internodes compared to low PAR (*p* ≤ 0.05, **Figure 3**). Overall, wild lentils had similar treatment responses compared to cultivated lentil for these characteristics.

Leaflet area at the R1 node had the same trend as shoot and internode length, being significantly influenced by genotype, the light environment, and the interaction between them (**Table 3**). The largest leaflet area resulted from growth in the low R/FR low PAR environment (*p* ≤ 0.05; **Figure 3**), except for *L. culinaris* CDC Greenstar for which a high R/FR environment with a high PAR resulted in larger leaflet area compared to a low R/FR with low PAR.

### Yield, above Ground Biomass, and Harvest Index of *Lens* Genotypes to Changes in Light Environment

**FIGURE 4:**
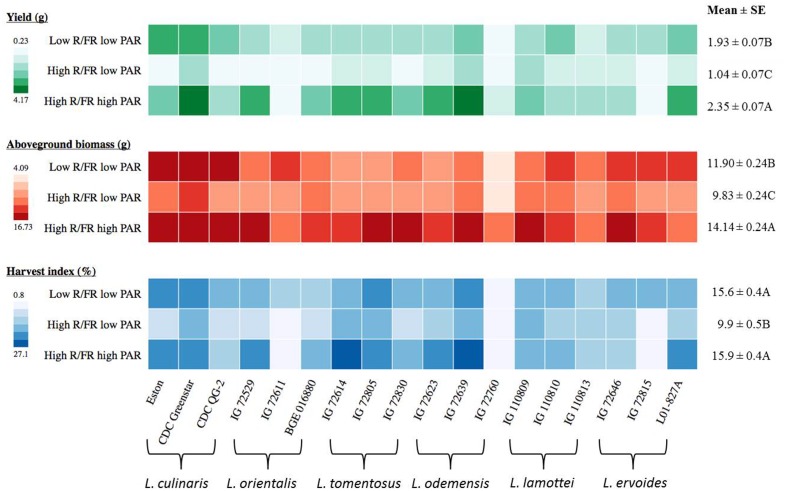
**Yield, above-ground biomass, and harvest index for three genotypes of each of six *Lens* species grown in light environments differing in red/far-red ratio (R/FR) and PAR.** Mean ± SE is from two repeats (eight replicates) for yield and one repeat (four replicates) for above-ground biomass and harvest index and it represents all *Lens* genotypes grown in the specific light environment. Different letters represent significant differences among treatments (Tukey’s test at *p* ≤ 0.05). Heat map is generated using the in-house developed computer program and the monochrome color coding system was used for each trait with the color from lighter to darker represented the values from lower to higher.

Both yield and above-ground biomass of *Lens* genotypes were significantly influenced by the different light treatments and both traits showed similar trends across the different treatments (**Table 3** and **Figure 4**). Overall, *Lens* genotypes had the highest yield and above-ground biomass under the high R/FR high PAR condition, while the low R/FR low PAR was intermediate and the high R/FR low PAR had the lowest (*p* ≤ 0.05, **Figure 4**). However, the yields of some wild lentil genotypes, including three *L. lamottei* genotypes (IG 110809, IG 110810, and IG 110813) and *L. ervoides* IG 72646, were not significantly different across all light treatments (*p* ≤ 0.05). The yields of genotypes *L. orientalis* IG 72611 and *L. ervoides* IG 72815 under the high R/FR condition were not representative of the true yields because the experiments were stopped at 110 days after sowing. The flowering time of these two genotypes were significantly delayed under the high R/FR environment, which therefore delayed both pod setting and maturity. For *L. odemensis* IG 72760, late emergence combined with a low PAR reduced seed yield.

The harvest index of *Lens* genotypes was significantly influenced by genotype, light environment, and the interaction between them (**Table 3**). Overall, the harvest indices of *Lens* genotypes were not significantly different (*p* ≤ 0.05) under low R/FR low PAR and high R/FR high PAR conditions, whereas the high R/FR low PAR condition resulted in a significant decrease (*p* ≤ 0.05, **Figure 4**). However, harvest indices of all three *L. lamottei* genotypes (IG 110809, IG 110810, and IG 110813), *L. ervoides* IG 72646, and *L. tomentosus* IG 72805 were not significantly different across all three light treatments (*p* ≤ 0.05). The harvest indices of *L. orientalis* IG 72611 and *L. ervoides* IG 72815 under the high R/FR condition were not representative of the true harvest indices because the experiments were stopped at 110 days after sowing and the delayed flowering of these two genotypes affected the yields as mentioned previously, therefore affected the harvest indices. The harvest index of *L. odemensis* IG 72760 was also not representative of the true harvest index due to affected yield from late emergence and low PAR as mentioned in previous yield section.

## Discussion

### Flowering Initiation of *Lens* Genotypes is Mainly Influenced by R/FR Related Light Quality Change

Plants depend on the acquisition of light energy for their survival, and competition for light is a characteristic of plant communities. Responses to changes in light quality and intensity enables plants to adapt and optimize their subsequent growth and development. A natural light environment under a canopy has a low R/FR ratio since plants absorb most of the visible light (from 400 to 700 nm) but reflect most of the FR light (Smith, 1982, 1994). A low R/FR ratio reflected from the surrounding vegetation may create a signal to plants of potential competition for light and, therefore, they initiate escape or SARs (Ballare et al., 1990). If the reduced R/FR ratio signal persists and the plant is unable to overcome competing vegetation by growth extension, flowering is accelerated, thereby promoting seed set and enhancing the probability of reproductive success (Halliday et al., 1994; Smith and Whitelam, 1997).

The current study tested seven species of *Lens* and demonstrated that only the cultivated lentil (*L. culinaris*) showed consistent responses to the low R/FR light treatment and this low R/FR light quality promoted early flowering. Most wild lentil genotypes exhibited reduced responses toward the light quality changes and flowering times were not significantly affected. Three wild lentil genotypes (*L. orientalis* IG 72611, *L. tomentosus* IG 72830, and *L. ervoides* IG 72815) had similar flowering responses to the cultivated lentil. However, a genotyping-by-sequencing (GBS) study of the genus *Lens* (Wong et al., 2015) concluded that these three lines fall outside the cluster of other members of those species and may be natural hybrids. This hints at the possibility of transferring the genes controlling the response from one species to another.

A general model for red and far-red light absorbing phytochrome action is that phytochromes perceive light, enter the nucleus, and then interact with transcriptional regulators to regulate gene transcription (Chen et al., 2004; Lorrain et al., 2006; Han et al., 2007). Five members of the phytochrome family were discovered in *Arabidopsis*, named phy A to phy E (Sharrock and Quail, 1989; Clack et al., 1994), which have differential photosensory and physiological roles in controlling plant growth and development (Smith and Whitelam, 1990; Whitelam and Devlin, 1997; Franklin and Quail, 2010). Some of the phytochrome functions elucidated through analysis of *Arabidopsis* mutants demonstrated that the suppression of flowering under a high R/FR ratio is mediated predominantly by phy B, with redundancy roles for phy D and phy E (Whitelam and Smith, 1991; Halliday et al., 1994; Devlin et al., 1999; Franklin et al., 2003). Genetically distinct signaling pathway segments among the phytochrome family members were also identified (Li et al., 2011). Various studies report that phytochrome genes and flowering genes are well-conserved between *Arabidopsis* and legumes at the level of sequence and physiological function (Hecht et al., 2005; Ueoka-Nakanishi et al., 2011; Wu et al., 2011). Therefore, we suspect that differences in genes of the red and far-red light absorbing phytochrome family and its signaling pathway may also play a direct and important role with respect to the different flowering responses within *Lens* genotypes. Through the domestication process, the variant(s) that make a plant more sensitive to R/FR may have been retained.

The spectral distribution in terms of R/FR ratio of daylight is broadly constant at a specific latitude but varies considerably with geographical location (Smith, 1982). Maloof et al. (2001) reported a correlation between light response and latitude of origin regarding the hypocotyl lengths in 141 *Arabidopsis thaliana* accessions, but no connection regarding the longitude. However, this may not be the case in current study where *Lens nigricans* was the only wild lentil species that showed a large response to change in light environment (no sign of flowering even 110 days after sowing) yet the latitude of origin for *L. nigricans* is well within the range of other *Lens* species. Previous experience (Saha, unpublished) and results from the current study suggest that favorable flowering conditions for *L. nigricans* may be the low R/FR high PAR condition, which was not assessed here due to limitations of the available lighting systems. A quaternary gene pool placement proposed by Wong et al. (2015) might explain the distinctly different responses of the *L. nigricans* group to the different light quality treatments compared to the other *Lens* species. This species might also have evolved to feature an extended juvenile phase.

### Vegetative Growth in *Lens* Genotypes was Mainly Affected by Light Quality with an Interaction of Light Intensity and Controlled by Discrete Pathways from Flowering

The strategy of low R/FR induced shade avoidance used by many plants is to promote growth extension in an attempt to harvest more available light (Runkle and Heins, 2001) and, therefore, the most dramatic SAR is the stimulation of elongation (Devlin et al., 1996; Morelli and Ruberti, 2000). This was clearly shown in our current study. Shoot length and internode length were the longest under the low R/FR environment in all *Lens* genotypes. Reductions in light intensity usually trigger SARs similar to those under a low R/FR condition (Ballare et al., 1991). In our study, however, we found that high PAR stimulated shoot and internode elongation in *Lens* genotypes in comparison to low PAR under the high R/FR environment, which might be due to the high assimilation rate and high source/sink ratio that occur in the high PAR environment. Evers et al. (2011) observed a similar result, where a high PAR condition promoted branch growth in *Arabidopsis*. Overall, our results show that a low R/FR environment promotes shoot and internode elongation in *Lens* genotypes and high PAR also contributes to elongation. To expand or elongate an organ, as found in a SAR, plants must have a coupling mechanism to process cell division, cell elongation, and cell differentiation. The combined action of plant hormones including gibberellin and auxin play an important role in coordinating the response (Morelli and Ruberti, 2000; Franklin, 2008). The distinctly different responses in flowering time and elongation under low R/FR conditions in the wild lentil species but not in the cultivated lentil suggest discrete pathways control flowering and elongation, both of which are components of the SAR. Separate signaling mechanisms were reported to operate downstream of phytochromes to regulate elongation and flowering responses to low R/FR in *Arabidopsis* (Franklin, 2008).

In the current study, leaflet area increased overall under the low R/FR environment, although with some exceptions. Baldissera et al. (2014) reported that shaded alfalfa plants have larger leaves; however, those of wild-type *Arabidopsis* seedlings display a leaf size decrease in response to low R/FR (Devlin et al., 1999). Increased leaf area under a low PAR environment has been observed in various plant species (Kremer and Kropff, 1999; James and Bell, 2000; Liao et al., 2006), and the common assumption is that increased leaf area will help increase light interception. Moreover, plant leaves grown under high PAR have lower photosynthetic pigment content than leaves grown under low PAR (Mielke and Schaffer, 2010). Under light deficit conditions, plants set a series of compensatory mechanisms, such as incremental increase of the photosynthetic pigment content, change of the leaf angle toward the light source, or an increase in leaf area, to achieve higher light absorption efficiency. The latter might be the case in most *Lens* genotypes in a low R/FR environment.

### Reproductive Growth in *Lens* Genotypes was Mainly Affected by Light Intensity with an Interaction of Light Quality

Light is the main source of energy for carbon assimilation and growth in plants, therefore yield and biomass reductions occur under reduced light intensity (Baligar et al., 2006; Polthanee et al., 2011). In our study, the overall yield and above-ground biomass were highest under the high PAR environment, which indicates reproductive growth in *Lens* genotypes is mainly affected by light intensity. Low R/FR induced SARs involve a marked redirection of assimilates toward elongation and away from structures dedicated to resource acquisition and storage in natural conditions that limit water and nutrient resources (Smith and Whitelam, 1997). For crop species, a SAR could lead to decreased yield if plants expend resources on vegetative growth at the expense of reproductive development (Kebrom and Brutnell, 2007; Casal, 2013). In the low R/FR indoor settings of our study, the SARs were clear in most *Lens* genotypes, resulting in reduced yield even though plants had sufficient water and nutrients. The *L. lamottei* group and *L. ervoides* IG 72646, however, maintained comparable yield, biomass, and harvest index under all three light environments, which may indicate better adaptation to changes in light environment. Maloof et al. (2001) reported natural variation in light sensitivity across a diverse set of *A. thaliana* accessions, and Hancock et al. (2011) detected and identified PAR-adaptive alleles in *A. thaliana* using a genome-wide scan. Identification of these light-adaptive alleles would further help on understanding of the genetic basis of light responses in lentil; such work is currently under way in our group.

## Conclusion

Differences in light quality and intensity will affect the growth and development patterns of *Lens* plants, although some species are less affected than others. The high R/FR ratio created by fluorescent bulbs is not uncommon in controlled environment growth chambers. The results suggest that caution should be exercised in controlled environment growth chambers because the spectral property of the artificial light sources could severely delay the flowering of some crops, such as lentil, and thereby cause mismatch and delay for indoor breeding cycles. The identification of some *Lens* species that were less sensitive to R/FR related light quality and PAR related light intensity change may indicate a better adaptability toward changes in light environment. These varied responses might represent a source of genetic diversity that could be deployed in cultivated lentil to allow it to better handle sub-optimal light environments. Overall, increased understanding of light responses will help improve our ability to develop cultivars that have better adaptation to variable light environments.

## Author Contributions

Conception and design of the experiments: AV, SS, HY, KB. Acquisition of the data: SS, HY. Analysis and interpretation of the data: HY, KB, AV, SS. Drafting and revision of the paper: HY, KB, AV, SS.

## Conflict of Interest Statement

The authors declare that the research was conducted in the absence of any commercial or financial relationships that could be construed as a potential conflict of interest.

## Footnotes

1 http://www.sungro.com/professional-products
