# Supplementary material for: Flowering and Growth Responses of Cultivated Lentil and Wild Lens Germplasm toward the Differences in Red to Far-Red Ratio and Photosynthetically Active Radiation
Source: Front Plant Sci. 2017 Mar 21;8:386. doi: 10.3389/fpls.2017.00386 (PMC5359283; doi:10.3389/fpls.2017.00386)
Supplement: Supplementary file 1 [file Data_Sheet_1.DOCX]

Supplementary Material

**Flowering and growth responses of cultivated lentil and wild *Lens* germplasm towards the differences in red/ far-red ratio and photosynthetically active radiation**

Hai Ying Yuan, Shyamali Saha, Albert Vandenberg, and Kirstin E. Bett*

*Correspondence: Dr. Kirstin E. Bett [k.bett@usask.ca](mailto:k.bett@usask.ca)

1. Supplementary Data

Supplementary data 1. Original data used to generate figure 2 heatmap on days to flower for three genotypes of each of six *Lens* species on light environments differing in red/far-red ratio (R/FR) and photosynthetic active radiation (PAR). Data represent Mean ± SE.

| Species | Genotypes | Low R/FR low PAR | High R/FR low PAR | High R/FR high PAR |
| --- | --- | --- | --- | --- |
| *L. culinaris* | Eston | 38±1.7 | 49±1.7 | 48±1.7 |
|  | CDC Greenstar | 38±1.7 | 44±1.7 | 45±1.7 |
|  | CDC QG-2 | 44±1.7 | 52±1.7 | 55±1.7 |
| *L. orientalis* | IG 72529 | 25±1.7 | 30±1.7 | 29±1.8 |
|  | IG 72611 | 30±1.7 | 45±1.7 | 48±1.7 |
|  | BGE 016880 | 29±1.7 | 33±1.7 | 34±1.7 |
| *L. tomentosus* | IG 72614 | 21±1.7 | 28±1.7 | 23±1.7 |
|  | IG 72805 | 25±1.7 | 28±1.7 | 29±1.7 |
|  | IG 72830 | 29±1.7 | 40±1.7 | 38±1.7 |
| *L. odemensis* | IG 72623 | 27±1.7 | 30±1.7 | 29±1.7 |
|  | IG 72639 | 22±1.7 | 29±1.7 | 29±1.7 |
|  | IG 72760 | 31±2.0 | 37±1.8 | 34±2.0 |
| *L. lamottei* | IG 110809 | 28±1.7 | 28±1.7 | 31±1.7 |
|  | IG 110810 | 25±1.7 | 30±1.7 | 30±1.7 |
|  | IG 110813 | 28±1.7 | 28±1.8 | 26±1.7 |
| *L. ervoides* | IG 72646 | 30±1.7 | 37±1.7 | 35±1.7 |
|  | IG 72815 | 34±1.8 | 47±1.7 | 49±1.7 |
|  | L01-827A | 21±1.7 | 23±1.7 | 25±1.7 |

Supplementary data 2. Original data used to generate figure 3 heatmap on shoot length for three genotypes of each of six *Lens* species on light environments differing in red/far-red ratio (R/FR) and photosynthetic active radiation (PAR). Data represent Mean ± SE.

| Species | Genotypes | Low R/FR low PAR | High R/FR low PAR | High R/FR high PAR |
| --- | --- | --- | --- | --- |
| *L. culinaris* | Eston | 39.3±2.0 | 29.2±2.0 | 33.4±2.0 |
|  | CDC Greenstar | 37.2±2.0 | 28.5±2.1 | 35.1±2.0 |
|  | CDC QG-2 | 35.4±2.0 | 26.2±2.0 | 30.9±2.0 |
| *L. orientalis* | IG 72529 | 30.8±2.0 | 23.2±2.0 | 27.8±2.1 |
|  | IG 72611 | 29.3±2.0 | 18.7±2.0 | 21.1±2.0 |
|  | BGE 016880 | 31.6±2.0 | 24.5±2.0 | 27.5±2.0 |
| *L. tomentosus* | IG 72614 | 29.7±2.0 | 17.0±2.0 | 20.3±2.0 |
|  | IG 72805 | 28.6±2.0 | 17.4±2.0 | 24.3±2.0 |
|  | IG 72830 | 38.5±2.0 | 24.7±2.1 | 26.6±2.0 |
| *L. odemensis* | IG 72623 | 29.4±2.0 | 19.0±2.0 | 21.4±2.0 |
|  | IG 72639 | 32.0±2.0 | 20.7±2.0 | 24.9±2.0 |
|  | IG 72760 | 28.0±2.5 | 21.7±2.3 | 24.1±2.3 |
| *L. lamottei* | IG 110809 | 43.3±2.0 | 21.6±2.1 | 30.5±2.0 |
|  | IG 110810 | 40.5±2.0 | 22.3±2.0 | 31.4±2.0 |
|  | IG 110813 | 42.0±2.0 | 23.5±2.1 | 33.3±2.0 |
| *L. ervoides* | IG 72646 | 36.6±2.0 | 24.9±2.0 | 28.2±2.0 |
|  | IG 72815 | 37.5±2.1 | 22.0±2.1 | 32.4±2.1 |
|  | L01-827A | 35.8±2.0 | 18.6±2.0 | 27.5±2.0 |

Supplementary data 3. Original data used to generate figure 3 heatmap on internode length for three genotypes of each of six *Lens* species on light environments differing in red/far-red ratio (R/FR) and photosynthetic active radiation (PAR). Data represent Mean ± SE.

| Species | Genotypes | Low R/FR low PAR | High R/FR low PAR | High R/FR high PAR |
| --- | --- | --- | --- | --- |
| *L. culinaris* | Eston | 2.27±0.12 | 1.63±0.12 | 1.89±0.12 |
|  | CDC Greenstar | 2.33±0.12 | 1.77±0.13 | 2.02±0.12 |
|  | CDC QG-2 | 2.01±0.12 | 1.44±0.12 | 1.64±0.12 |
| *L. orientalis* | IG 72529 | 2.17±0.12 | 1.64±0.12 | 1.89±0.13 |
|  | IG 72611 | 1.93±0.12 | 1.23±0.12 | 1.48±0.12 |
|  | BGE 016880 | 2.13±0.12 | 1.65±0.12 | 1.79±0.12 |
| *L. tomentosus* | IG 72614 | 1.85±0.12 | 1.05±0.12 | 1.25±0.12 |
|  | IG 72805 | 1.95±0.12 | 1.18±0.12 | 1.65±0.12 |
|  | IG 72830 | 2.17±0.12 | 1.38±0.13 | 1.60±0.12 |
| *L. odemensis* | IG 72623 | 1.79±0.12 | 1.23±0.12 | 1.43±0.12 |
|  | IG 72639 | 2.24±0.12 | 1.58±0.12 | 1.87±0.12 |
|  | IG 72760 | 1.93±0.15 | 1.42±0.14 | 1.69±0.14 |
| *L. lamottei* | IG 110809 | 2.22±0.12 | 1.24±0.13 | 1.54±0.12 |
|  | IG 110810 | 2.25±0.12 | 1.36±0.12 | 1.78±0.12 |
|  | IG 110813 | 2.26±0.12 | 1.32±0.13 | 1.69±0.12 |
| *L. ervoides* | IG 72646 | 2.37±0.12 | 1.68±0.12 | 2.00±0.12 |
|  | IG 72815 | 2.35±0.13 | 1.41±0.13 | 1.98±0.13 |
|  | L01-827A | 2.20±0.12 | 1.35±0.12 | 2.06±0.12 |

Supplementary data 4. Original data used to generate figure 3 heatmap on leaflet area for three genotypes of each of six *Lens* species on light environments differing in red/far-red ratio (R/FR) and photosynthetic active radiation (PAR). Data represent Mean ± SE.

| Species | Genotypes | Low R/FR low PAR | High R/FR low PAR | High R/FR high PAR |
| --- | --- | --- | --- | --- |
| *L. culinaris* | Eston | 0.29±0.02 | 0.15±0.02 | 0.19±0.02 |
|  | CDC Greenstar | 0.20±0.02 | 0.13±0.02 | 0.23±0.02 |
|  | CDC QG-2 | 0.24±0.02 | 0.20±0.02 | 0.21±0.02 |
| *L. orientalis* | IG 72529 | 0.17±0.02 | 0.08±0.02 | 0.15±0.02 |
|  | IG 72611 | 0.23±0.02 | 0.10±0.02 | 0.18±0.02 |
|  | BGE 016880 | 0.26±0.02 | 0.25±0.02 | 0.30±0.02 |
| *L. tomentosus* | IG 72614 | 0.14±0.02 | 0.07±0.02 | 0.08±0.02 |
|  | IG 72805 | 0.16±0.02 | 0.08±0.02 | 0.11±0.02 |
|  | IG 72830 | 0.17±0.02 | 0.09±0.02 | 0.16±0.02 |
| *L. odemensis* | IG 72623 | 0.12±0.02 | 0.08±0.02 | 0.11±0.02 |
|  | IG 72639 | 0.10±0.02 | 0.06±0.02 | 0.10±0.02 |
|  | IG 72760 | 0.18±0.02 | 0.05±0.02 | 0.15±0.02 |
| *L. lamottei* | IG 110809 | 0.19±0.02 | 0.13±0.02 | 0.17±0.02 |
|  | IG 110810 | 0.18±0.02 | 0.17±0.02 | 0.11±0.02 |
|  | IG 110813 | 0.14±0.02 | 0.11±0.02 | 0.10±0.02 |
| *L. ervoides* | IG 72646 | 0.23±0.02 | 0.14±0.02 | 0.13±0.02 |
|  | IG 72815 | 0.18±0.02 | 0.13±0.02 | 0.14±0.02 |
|  | L01-827A | 0.16±0.02 | 0.08±0.02 | 0.12±0.02 |

Supplementary data 5. Original data used to generate figure 4 heatmap on yield for three genotypes of each of six *Lens* species on light environments differing in red/far-red ratio (R/FR) and photosynthetic active radiation (PAR). Data represent Mean ± SE.

| Species | Genotypes | Low R/FR low PAR | High R/FR low PAR | High R/FR high PAR |
| --- | --- | --- | --- | --- |
| *L. culinaris* | Eston | 3.00±0.27 | 0.87±0.27 | 2.82±0.27 |
|  | CDC Greenstar | 3.45±0.27 | 1.96±0.31 | 3.66±0.27 |
|  | CDC QG-2 | 2.62±0.27 | 0.60±0.31 | 2.17±0.27 |
| *L. orientalis* | IG 72529 | 1.69±0.27 | 0.82±0.27 | 3.11±0.38 |
|  | IG 72611 | 1.44±0.31 | 0.28±0.27 | 0.49±0.27 |
|  | BGE 016880 | 1.62±0.31 | 0.86±0.31 | 2.38±0.31 |
| *L. tomentosus* | IG 72614 | 1.75±0.27 | 1.36±0.27 | 3.37±0.27 |
|  | IG 72805 | 1.92±0.27 | 1.42±0.27 | 3.29±0.27 |
|  | IG 72830 | 1.74±0.27 | 0.70±0.27 | 2.43±0.27 |
| *L. odemensis* | IG 72623 | 1.74±0.27 | 1.01±0.27 | 2.87±0.27 |
|  | IG 72639 | 2.50±0.27 | 1.98±0.27 | 4.17±0.27 |
|  | IG 72760 | 0.29±0.31 | 0.30±0.31 | 1.15±0.38 |
| *L. lamottei* | IG 110809 | 1.84±0.27 | 1.70±0.38 | 2.23±0.27 |
|  | IG 110810 | 2.22±0.27 | 1.48±0.27 | 2.17±0.27 |
|  | IG 110813 | 1.53±0.27 | 1.20±0.31 | 1.74±0.27 |
| *L. ervoides* | IG 72646 | 1.99±0.27 | 1.41±0.31 | 1.99±0.31 |
|  | IG 72815 | 1.95±0.31 | 0.24±0.38 | 0.23±0.27 |
|  | L01-827A | 2.31±0.27 | 0.94±0.27 | 3.09±0.31 |

Supplementary data 6. Original data used to generate figure 4 heatmap on above-ground biomass for three genotypes of each of six *Lens* species on light environments differing in red/far-red ratio (R/FR) and photosynthetic active radiation (PAR). Data represent Mean ± SE.

| Species | Genotypes | Low R/FR low PAR | High R/FR low PAR | High R/FR high PAR |
| --- | --- | --- | --- | --- |
| *L. culinaris* | Eston | 15.33±0.92 | 10.64±0.92 | 14.75±0.92 |
|  | CDC Greenstar | 15.34±0.92 | 13.25±1.07 | 16.73±0.92 |
|  | CDC QG-2 | 14.67±0.92 | 9.76±0.92 | 16.25±0.92 |
| *L. orientalis* | IG 72529 | 10.47±0.92 | 9.45±0.92 | 15.91±1.07 |
|  | IG 72611 | 13.29±0.92 | 8.48±0.92 | 10.83±0.92 |
|  | BGE 016880 | 11.65±0.92 | 11.59±1.07 | 13.34±0.92 |
| *L. tomentosus* | IG 72614 | 9.59±0.92 | 8.95±0.92 | 12.84±0.92 |
|  | IG 72805 | 10.38±0.92 | 9.54±0.92 | 15.86±0.92 |
|  | IG 72830 | 11.78±0.92 | 10.01±0.92 | 16.37±0.92 |
| *L. odemensis* | IG 72623 | 9.76±0.92 | 9.24±0.92 | 13.89±0.92 |
|  | IG 72639 | 11.77±0.92 | 10.96±0.92 | 15.30±0.92 |
|  | IG 72760 | 4.09±1.31 | 5.23±0.92 | 10.43±1.07 |
| *L. lamottei* | IG 110809 | 11.73±0.92 | 11.04±1.31 | 15.06±0.92 |
|  | IG 110810 | 12.70±0.92 | 11.96±0.92 | 13.16±0.92 |
|  | IG 110813 | 11.54±0.92 | 9.78±1.07 | 12.50±0.92 |
| *L. ervoides* | IG 72646 | 13.66±0.92 | 12.42±1.07 | 14.80±0.92 |
|  | IG 72815 | 13.61±1.07 | 9.16±0.92 | 12.66±0.92 |
|  | L01-827A | 12.79±0.92 | 8.86±0.92 | 12.25±0.92 |

Supplementary data 7. Original data used to generate figure 4 heatmap on harvest index for three genotypes of each of six *Lens* species on light environments differing in red/far-red ratio (R/FR) and photosynthetic active radiation (PAR). Data represent Mean ± SE.

| Species | Genotypes | Low R/FR low PAR | High R/FR low PAR | High R/FR high PAR |
| --- | --- | --- | --- | --- |
| *L. culinaris* | Eston | 19.6±1.6 | 7.8±1.6 | 19.3±1.6 |
|  | CDC Greenstar | 22.5±1.6 | 14.7±1.8 | 21.6±1.6 |
|  | CDC QG-2 | 17.7±1.6 | 6.2±1.8 | 13.3±1.6 |
| *L. orientalis* | IG 72529 | 16.6±1.6 | 8.7±1.6 | 19.4±2.2 |
|  | IG 72611 | 11.7±1.8 | 3.3±1.6 | 4.3±1.6 |
|  | BGE 016880 | 12.7±1.8 | 7.1±1.8 | 16.5±1.8 |
| *L. tomentosus* | IG 72614 | 18.3±1.6 | 15.2±1.6 | 25.8±1.6 |
|  | IG 72805 | 18.6±1.6 | 15.1±1.6 | 20.5±1.6 |
|  | IG 72830 | 14.8±1.6 | 7.1±1.6 | 14.9±1.6 |
| *L. odemensis* | IG 72623 | 17.7±1.6 | 10.8±1.6 | 20.8±1.6 |
|  | IG 72639 | 21.3±1.6 | 18.2±1.6 | 27.1±1.6 |
|  | IG 72760 | 0.8±3.1 | 2.2±3.1 | 1.8±3.1 |
| *L. lamottei* | IG 110809 | 15.8±1.6 | 15.3±2.2 | 14.7±1.6 |
|  | IG 110810 | 17.4±1.6 | 12.3±1.6 | 16.6±1.6 |
|  | IG 110813 | 13.3±1.6 | 12.2±1.8 | 13.9±1.6 |
| *L. ervoides* | IG 72646 | 14.5±1.6 | 11.2±1.8 | 12.9±1.8 |
|  | IG 72815 | 14.4±1.8 | 2.3±2.2 | 1.7±1.6 |
|  | L01-827A | 17.9±1.6 | 10.8±1.6 | 22.4±1.8 |

2. Supplementary Figures and Tables

2.1 Supplementary Figures


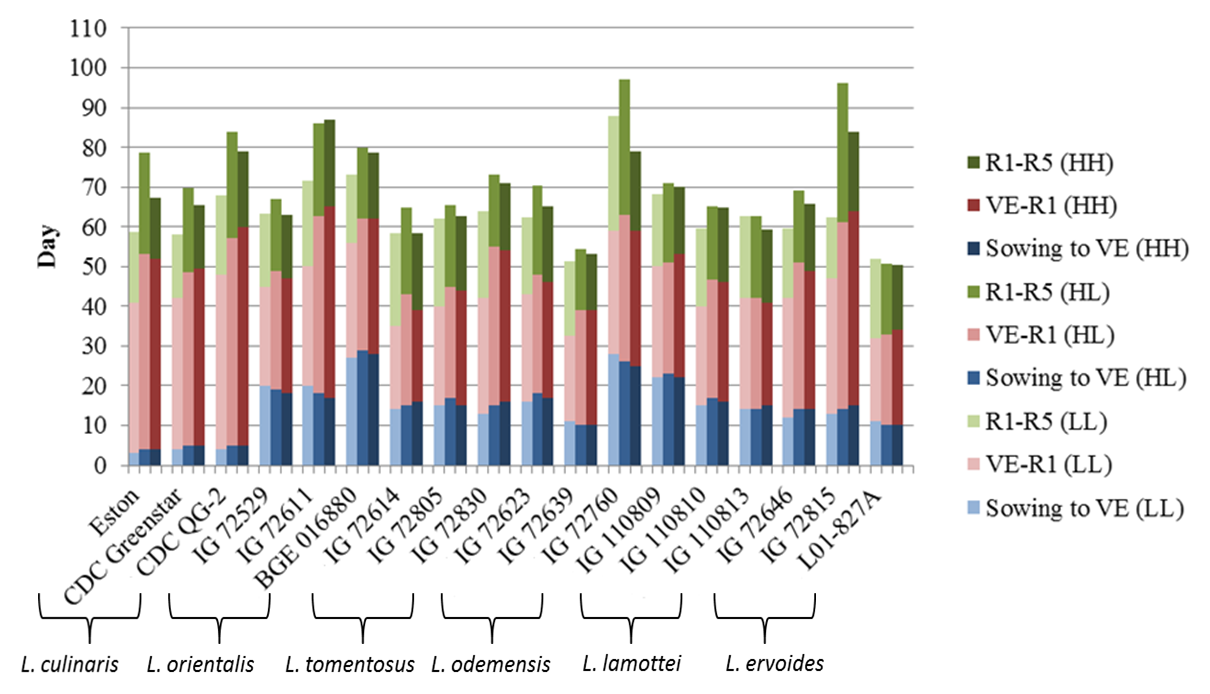


Supplementary Figure 1. Development stage partitioning of *Lens* genotypes grown under light environments differing in red/far-red ratio (R/FR) and photosynthetic active radiation (PAR). Data represent average from 2 repeats for each genotype. In figure legend VE means emergence, R1 means one open flower at any node, and R5 means one mature pod at any node. LL = low R/FR low PAR, HL = high R/FR high PAR and HH = high R/FR high PAR.

2.2 Supplementary Tables

Supplementary Table 1. Pearson correlation coefficients for days to flower, shoot length, internode length, above-ground biomass, yield and harvest index evaluated under light environments differing in red/far-red ratio (R/FR) and photosynthetically active radiation (PAR). *, **, *** indicates significant correlation at P ≤ 0.05, 0.01, 0.001, respectively, and ns = non-significant correlation.

| Traits | Shoot length | Internode length | Above-ground Biomass | Yield | Harvest index |
| --- | --- | --- | --- | --- | --- |
| Days to flower | -0.03^ns^ | -0.12^*^ | 0.03^ns^ | -0.14^*^ | -0.39^***^ |
| Shoot length |  | 0.73^***^ | 0.44^***^ | 0.27^***^ | 0.19^**^ |
| Internode length |  |  | 0.30^***^ | 0.14^*^ | 0.31^***^ |
| Above-ground Biomass |  |  |  | 0.68^***^ | 0.40^***^ |
| Yield |  |  |  |  | 0.92^***^ |
